# Management and Clinical Outcome of Aortic Graft Infections: A Single-Center Retrospective Study

**DOI:** 10.3390/jcm11216588

**Published:** 2022-11-07

**Authors:** Jinting Ge, Chengxin Weng, Jichun Zhao, Ding Yuan, Bin Huang, Tiehao Wang

**Affiliations:** Department of Vascular Surgery, West China Hospital, Sichuan University, 37 Guo Xue Alley, Chengdu 610041, China

**Keywords:** aortic graft infection, in-situ reconstruction, extra-anatomic reconstruction, conservative treatment

## Abstract

Background: This study aimed to evaluate the outcome of various treatment options for aortic graft infection (AGI) patients and identify factors affecting their prognosis. Methods: The data of AGI patients from January 2008 to December 2019 were retrospectively collected and analyzed. The primary endpoints were 30-day mortality and perioperative complication-related morbidity; the secondary endpoints were re-infection (RI) rates, primary and secondary graft patency, overall mortality, duration of antibiotic therapy, and the number of antibiotic types used in treatment. Results: There was no significant difference in the 30-day mortality and perioperative-related complications between the conservative treatment, in-situ reconstruction (ISR), and extra-anatomic reconstruction (EAR) groups. The ISR group had lower re-infection rates and better overall survival rates than the EAR and conservative treatment groups. Different bypass graft conduits had no significant influence on the RI rate or primary and secondary graft patency. AGI patients infected with high-virulence pathogens had higher RI and overall mortality rates than those infected with low virulence pathogens, but this was not statistically significant. Initial procedures prior to the AGI also had no influence on the prognosis of AGI patients. Patients undergoing ISR or EAR surgery received antibiotic therapy for a longer duration than patients undergoing conservative treatment. Patients without RI received more types of antibiotics than patients with RI. Conclusions: ISR had lower RI rates and better overall survival rates than EAR and conservative treatment and may be a better choice for patients with AGI. Several factors were found to have no influence on patients’ prognosis however, further studies are required.

## 1. Introduction

Aortic graft infections (AGIs) are rare but can lead to catastrophic consequences. The use of vascular procedures, particularly endovascular procedures, has rapidly increased in recent years. Therefore, the incidence of aortic graft infections (AGIs) is increasing, with 1–6% of patients who undergo vascular procedures suffering from AGIs. AGI is associated with high mortality (8–75%), major amputation (0–29%), aortic stump blowout (10–20%), and a high re-infection rate (5–40%) [1,2,3,4,5,6,7].

Although the European Society for Vascular Surgery (ESVS) published clinical practice guidelines for vascular graft and endograft infection (VGEI) in 2020, studies on the management of patients with AGI are still limited [8]. Furthermore, most published studies did not provide long-term follow-up results and did not have sufficient statistical power to identify factors that influence prognosis due to the small number of enrolled AGI patients.

Our study used data from a 12-year period and provided short-term and long-term follow-up results for AGI patients. It aimed to evaluate the various treatment options and identify the factors affecting the prognosis of AGI patients after primary treatment of their vascular diseases. Furthermore, our study innovatively evaluated the relationship between the duration of antibiotic therapy, the types of antibiotics used in the treatment, and the prognosis of AGI patients.

## 2. Materials and Methods

### 2.1. Study Population and Diagnostic Criteria of AGIs 

**Figure 1:**
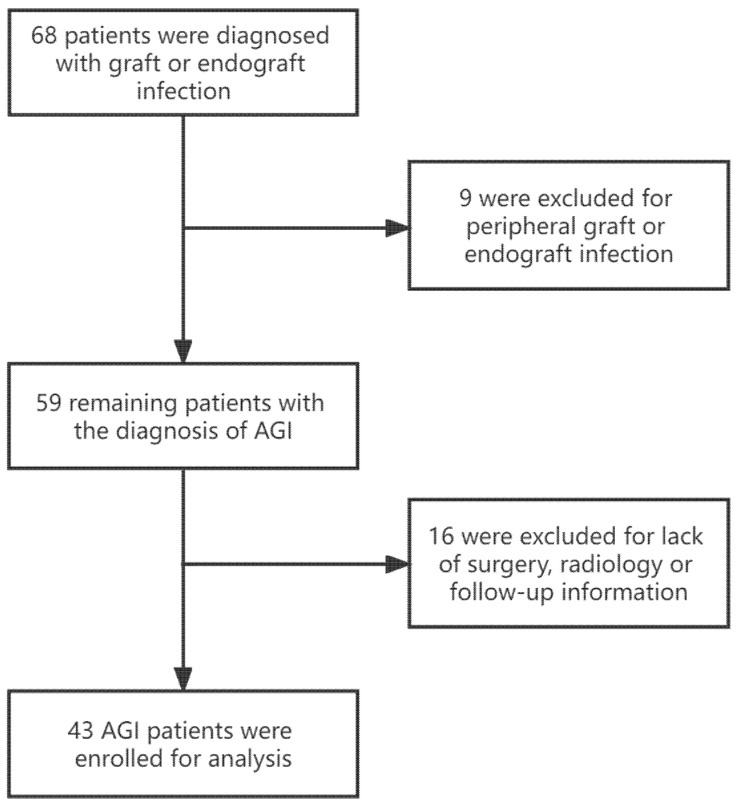
Flowchart of patients enrolled in the study.

**Figure 2:**
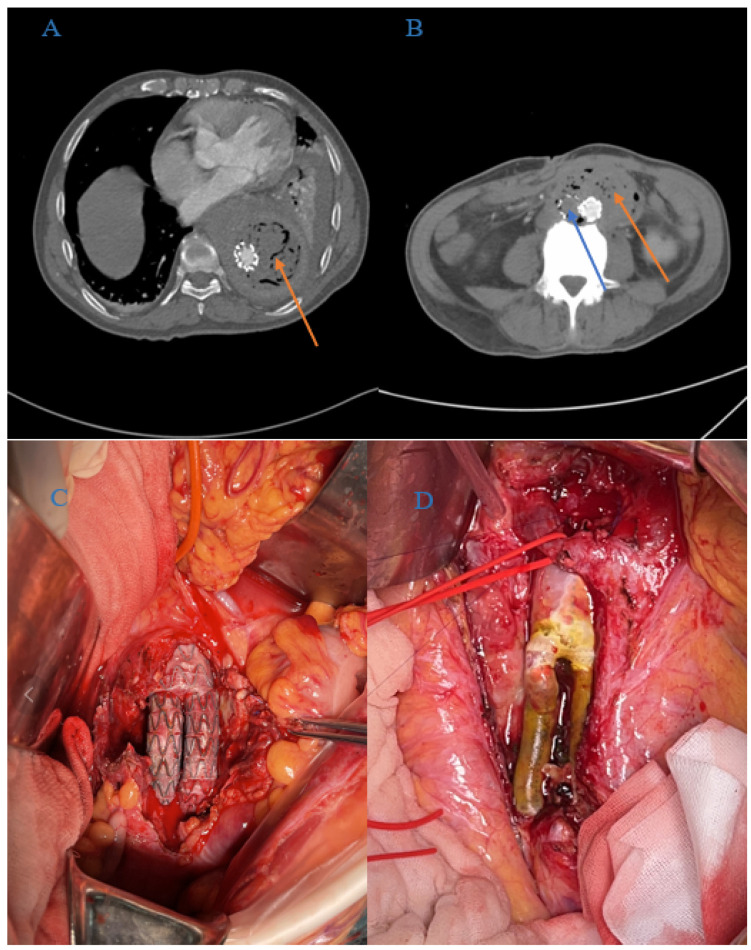
Peri-operative images of AGI patients. (**A**) Pre-operative CTA image of an AGI patient who previously underwent TEVAR (the orange arrow indicates the peri-graft fluid and gas). (**B**) Pre-operative CTA image of an AGI who underwent EVAR prior to AGI (orange and blue arrow indicate the peri-graft fluid and gas and graft thrombosis, respectively). (**C**) Peri-operative image of the ISR procedure. (**D**) Autologous vein was used as bypass graft conduits in ISR procedures.

From January 2008 to December 2019, 43 patients with AGI were admitted to our department (median age, 70 years; interquartile range, 57–78 years); their medical records were retrospectively collected from the hospital database for statistical analysis (see Figure 1). AGI was diagnosed based on clinical symptoms (fever, abdominal or back pain, gastrointestinal bleeding, dyspnea or hypoxemia, and so forth) and auxiliary examination results (radiography, ultrasonography, and CT angiography), which indicated the presence of peri-graft gas or fluid, retroperitoneal abscess, graft duodenal fistula, pseudoaneurysms, and graft thrombosis (see Figure 2A,B).

### 2.2. Peri-Operative Management and Operation Methods

The treatment regimen for patients with AGI was devised according to the patients’ general status, clinical symptoms, and auxiliary examination results. For those AGI patients who had insufficient etiological evidence, empirical antibiotics (usually piperacillin-tazobactam) were prescribed once they were admitted. The blood samples and abscess specimens were collected for microbiological culture whenever possible after admission to identify pathogenic bacteria and administer appropriate anti-infection therapy before the surgery. The anti-infection therapy of AGI patients would change according to their microbiological culture and drug susceptibility test results. The most common antibiotics for AGI patients include vancomycin, imipenem, and amikacin. Furthermore, the clinical course and variety of antibiotics would adjust according to the opinion of the infectious disease specialist. Conservative treatment with percutaneous drainage and irrigation was indicated for AGI patients with poor general status (severe anemia, hypoproteinemia, malnutrition, e.g.,) or serious pre-operative complications (such as advanced malignant diseases). Furthermore, conservative treatment may also be considered for certain AGI patients, such as thoracic/abdominal vascular graft/endograft infection patients with a high risk of surgery or thoracic vascular graft/endograft infection patients with no positive microbiological results, and an absence of an esophagus, the presence of an airway fistula, or severe sepsis. ISR was indicated as the first-line treatment for thoracic and abdominal AGI patients, especially when the infection is limited or the infected pathogen is a low-virulence strain, while EAR would be considered as an alternative option for patients with a large abscess or multi-resistant microorganisms. The infected stent grafts or vascular grafts would be completely removed, while an autologous vein, rifampicin-soaked ePTFE vascular grafts (600 mg rifampicin in 10–15 mL normal saline for 15–20 min at room temperature; Gore-Tex, W.L. Gore & Associates, Flagstaff, AZ, USA) or normal ePTFE vascular grafts were used as bypass conduits in ISR and ESR procedures (see Figure 2C,D). As for the indication of bypass graft conduits, an autologous vein was the first choice as the bypass conduit for AGI patients, especially for those with limited infection or low-virulence pathogens. Rifampicin-soaked ePTFE vascular graft was indicated for patients with high-virulence pathogens, large or multifocal abscesses, and generalized sepsis, while normal ePTFE vascular graft was indicated for patients without complex infection or multi-resistant pathogens, but autologous vein was not available. Autologous veins were mostly used in abdominal AGI patients because of their limited diameter; however, they can also be used as bypass grafts for TEVAR infection patients under certain conditions. In our cohort, one patient received TEVAR because of a previous thoracic aorta-arteritis pseudoaneurysm. The infected stent graft was removed during the surgery; the diameter of the infected aorta was smaller than normal thoracic aorta due to the affected section being in the distal end of the thoracic aorta, and the patient being diagnosed with aorta-arteritis, hence autologous vein was used as a bypass graft conduit in this patient. In addition, bilateral superficial femoral veins would usually be harvested and sutured together to increase the diameter of the bypass conduit in case of diameter mismatch; furthermore, we would split the femoral vein off and use spiral type anastomosis to re-suture it in order to increase the diameter of the autologous vein bypass graft further.

Due to the possibility that an autologous vein may be used as the conduit for bypass, all AGI patients received lower limb vein ultrasound examinations to evaluate the patency and reflux of veins in their lower extremities. The superficial femoral vein was the most common autologous vein used in the ISR or EAR procedures. In addition, for certain thoracic stent graft infection patients whose CTA or other auxiliary examinations indicated the possibility of a thoracic aorto-bronchial fistula (ABF) or an aorto-esophageal fistula(AEsF), broncho- or gastro-endoscope was used to confirm whether the fistula existed. Similarly, AGI patients who previously received EVAR or a AAA open repair and who have a suspicious aorto-enteric fistula (AEnF) would undergo gastrointestinal endoscopy to detect the existence of a fistula.

For thoracic aortic stent graft infection patients who received ISR surgery, autologous tissues (muscles or pleura) were used to cover the newly implanted graft. This is in contrast to patients who had undergone EAR surgery and suffered a thoracic aortic stent graft infection, where autologous tissues were used to cover the distal end of the thoracic aorta. A specialist in cardiothoracic surgery or gastrointestinal surgery would be consulted about surgical options if there was evidence of an aorto-bronchial fistula (ABF), an aorto-esophageal fistula (AEsF), or an aorto-enteric fistula (AEnF). Direct closure of the fistula would usually be considered for AGI patients with an ABF or an AEsF, while intestinal resection would be conducted for AGI patients with an AEnF. Furthermore, the ends ABF, AEsF, and AEnF were repaired by autologous tissues. In addition, emergency stent graft implantation would be considered a temporary measure in the event of active bleeding complicating an AGI with or without an ABF, AEsF, or AEnF.

### 2.3. Post-Operative Treatment and Follow-Up Management

Additional specimens and infected vascular stents or stent grafts were collected for bacterial culture and other investigations. The infected region was irrigated with antibiotics and diluted in povidone-iodine and normal saline during the surgery. All patients continued to receive treatment against infection after the surgery, and oral antibiotics were administered for 6 months or more, depending on the results of the microbiological culture, clinical symptoms, and the infectious disease physician’s assessment at the hospital.

**Figure 3:**
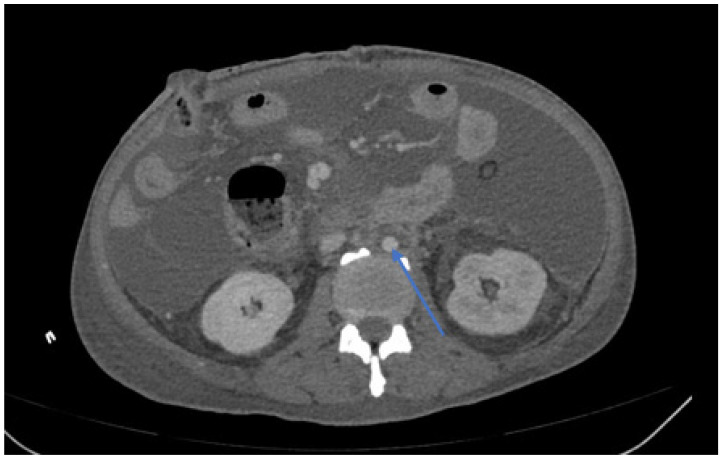
Post-operative CTA image of an abdominal stent graft infection in patients who underwent ISR and subsequent stent graft removal. CTA image at 6 months after ISR surgery indicated that the autologous vein graft was patent (blue arrow).

All patients were advised to return to the clinic at 3 and 6 months, and annually thereafter for further follow-up examinations. Patients underwent physical examinations for arterial pulse, routine blood tests, C-reactive protein (CRP), procalcitonin (PCT), interleukin-6 (IL-6) levels, and CTA to evaluate the outcome of treatment and determine further treatment plans during the follow-up clinical examinations (see Figure 3).

### 2.4. Primary and Secondary Endpoints

The primary endpoints of our study were 30-day mortality and perioperative complication-related morbidity. The secondary endpoints were re-infection (RI) rates, primary and secondary graft patency, overall mortality, duration of antibiotic therapy, and the total number of antibiotic types used for the treatment.

### 2.5. Statistical Analysis and Ethics Approval

To analyze differences between the means, a student *t*-test, one-way analysis of variance, and Dunnett-T3 test were performed. The Pearson chi-square test or Fisher exact test was used for the analysis of categorical variables. Overall survival and RI-free survival were assessed using Kaplan–Meier curves, and differences were analyzed using the log-rank test. A *p* < 0.05 indicated statistical significance. All statistical analyses were performed using the SPSS 21.0 software (IBM SPSS, New York, NY, USA).

This study was approved by the Ethics Committee of West China Hospital of Sichuan University (No.2021-150). Informed consent was obtained from the patients and their family.

## 3. Results

### 3.1. Baseline Characteristics

From January 2008 to December 2019, 43 consecutive patients received the diagnosis of AGI at the West China Hospital of Sichuan University. The mean follow-up duration of these patients was 45.9 ± 32.4 months (range, 3–115 months). Of these, 31 patients (72%) had hypertension; other pertinent risk factors included smoking or a history of smoking (25, 58%), diabetes mellitus (17, 39%), chronic obstructive pulmonary disease (11, 25%), hypoproteinemia (10, 23%), coronary artery disease (6, 13%), and chronic kidney failure (5, 11%). Sepsis, fever, leukocytosis, and bacteremia were the most common clinical symptoms. Of the 43 patients, 29 (67%) presented with sepsis on admission. Other common symptoms included abdominal or back pain, retroperitoneal abscess, graft duodenal fistula, acute major bleeding, lower-limb ischemia, and pseudoaneurysms. The mean time between treatment of initial vascular disease and diagnosis of VGEIwas 27.7 ± 19.6 months (range, 1–97 months). Detailed information about baseline and clinical symptoms are summarized in Table 1.

Table 2 summarizes the initial treatment for patients’ pre-existing vascular diseases before they were diagnosed with AGI. Of all 43 AGI patients, 18 (42%) received endovascular aortic aneurysm repair (EVAR), 13 (30%) patients received thoracic endovascular aortic aneurysm repair (TEVAR), while another 10 (23%) patients underwent abdominal aortic aneurysm (AAA) open repair, and finally, 2 (5%) patients received carotid-subclavian artery bypass because the left subclavian artery (LSA) was affected by the dissected aorta.

The most common pathogenic bacterium was *Staphylococcus aureus*, which was identified in 15 patients (34%). The other gram-positive bacteria identified included methicillin-resistant *Staphylococcus aureus* (MRSA) in 4 (9%), *Streptococcus* in 3 (6%), and *Enterococcus* in 3 patients (6%). *Escherichia coli* was the most frequent gram-negative bacterium (12, 27%). Other gram-negative bacteria identified were *Klebsiella pneumoniae*, *Pseudomonas aeruginosa*, *Enterobacter cloacae*, and *Salmonella* spp. Two patients also had a fungal infection. A polymicrobial infection was seen in 17 patients (39%). Blood cultures or specimen cultures were negative in 6 patients (13%). Multi-drug resistant bacteria, such as MRSA, was defined as high virulence pathogens. The blood culture results revealed that 11 of 43 AGI patients were infected with high virulence pathogens, while the other 32 AGI patients were infected with low virulence pathogens. There was no significant difference in the high virulence infection rate in AGI patients who had previously received endovascular or open surgery (8/33 vs. 3/10, *p* = 0.715). Table 3 lists the various bacteria cultured.

Seven patients (16%) received conservative treatment (antibiotics with percutaneous drainage and irrigation) because of their poor general status, negative auxiliary examination results, or advanced malignant diseases. These patients had poor prognoses: four patients had an RI, and three were re-admitted to our hospital for ISR or EAR. All remaining patients (39, 90%) received reconstruction surgery and complete graft removal, including ISR in 29 patients and EAR in 10 patients. The mean surgery time was 5.8 ± 4.4 h (range, 4–13 h). Specifically, of all 29 patients who received ISR surgery, 21 of them underwent AAA endovascular or open repair previously (15 EVAR and 6 open repair), while 8 of them underwent TEVAR (with or without carotid-subclavian artery bypass) prior to the diagnosis of AGI. As for patients who underwent EAR surgery, six of them received AAA repair (three EVAR and three open repair), and the other four patients underwent TEVAR before they were diagnosed with AGI. It is worth emphasizing that the surgery method for AGI patients who previously received TEVAR plus carotid-subclavian artery bypass was determined according to the pre-operative examinations and intraoperative situation. If the bypass graft of carotid-subclavian artery was affected by the AGI, we would completely remove the infected bypass graft and ligate the LSA. We would also use vasodilator medications, and monitor the left upper extremity after ligating the LSA, and decide whether to perform a secondary LSA bypass surgery based on the blood supply situation.

Furthermore, 11 patients (25%) received an AEnF repair and 4 patients (9%) received ABF or AesF repairs alongside an ISR or an EAR procedure. Three patients (6%) also received stent graft implantation as a temporary treatment measure because of the major bleeding caused by AGI. All implanted stent grafts were removed during the ISR or the EAR procedures.

### 3.2. Outcomes

#### 3.2.1. Early Mortality and Complication-Related Morbidity

The overall 30-day mortality rate was 25% (11/43),. Mortality was 17% (5/29), 30% (3/10), and 42% (3/7) in the ISR, EAR, and conservative treatment groups, respectively. The most frequent cause of mortality was respiratory failure (n = 4), other common causes of mortality were septic shock (n = 3), acute myocardial infarction (n = 1), major bleeding (n = 1), multiple organ dysfunction syndrome (n = 1), and unknown cause (n = 1).

No significant differences in 30-day mortality were seen among the conservative treatment, ISR, and ESR groups. The factors affecting 30-day mortality per the univariate analysis were: the presence of a statistically significant difference between elective and emergency procedures and a peak PCT level of >1 ng/mL during the hospital stay (*p* < 0.05, see Table 4). However, multivariate analysis revealed that only emergency procedures were associated with 30-day mortality (*p* < 0.05), while peak PCT level had no influence on 30-day mortality (see Table 5).

The average length of hospital stay was 28.9 ± 16.4 days (range, 4–82 days). In total, 19 patients (44%) experienced perioperative complications: 14 in the ISR group and. 5 in the EAR group (*p* = 1.00). Respiratory complications (n = 6) were the most common, which could be associated with the prolonged bedridden condition of the patients. Five patients had renal failure, two needed temporary dialysis, and one needed permanent dialysis. Other perioperative complications were lower limb edema due to venous insufficiency (n = 4), wound infection (n = 3), deep vein thrombosis (DVT; n = 2), heart failure (n = 2), multiple organ dysfunction syndrome (n = 2), major bleeding (n = 1), gastrointestinal bleeding (n = 1), paraplegia (n = 1), and cerebral infarction (n = 1). Seven patients experienced more than one complication.

Nine patients had to undergo a secondary surgery because of perioperative complications. Five patients were treated with thrombectomy, catheter-directed thrombolysis, or femoral-popliteal bypass procedures because of acute limb ischemia caused by graft occlusion. One in five patients with ischemia underwent an amputation because of irreversible ischemia. Two patients with bleeding underwent a repeat abdominal exploration surgery. One patient with DVT received a vena cava filter implantation, and one patient with acute myocardial infarction underwent percutaneous transluminal coronary angioplasty and coronary artery stent implantation.

#### 3.2.2. RI Rate

**Figure 4:**
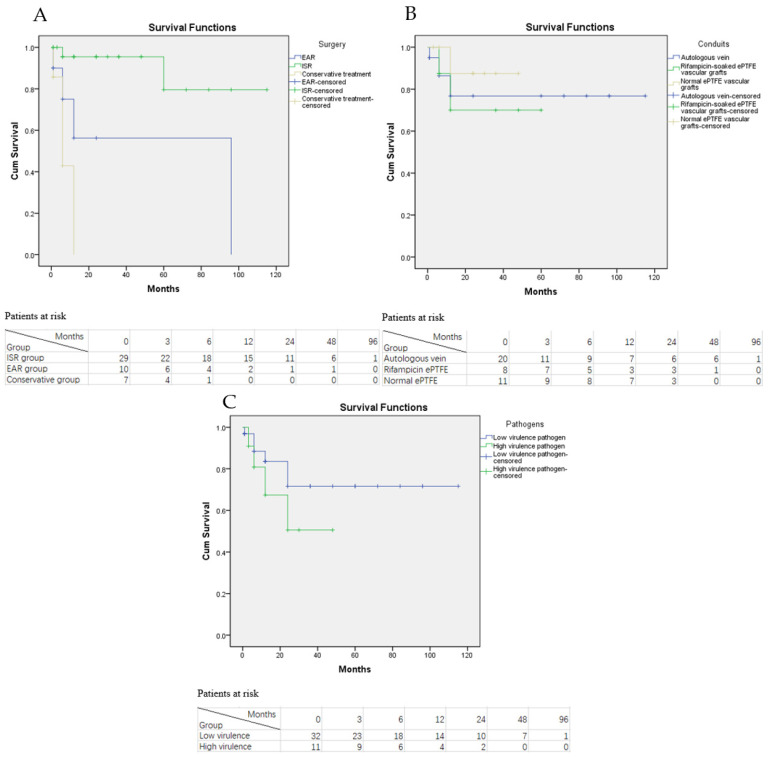
Kaplan-Meier curve of the RI rate in AGI patients. (**A**) KM curve of the RI rate grouped by treatment option with the numbers of patients at risk; (**B**) KM curve of the RI rate grouped by bypass graft conduits with the numbers of patients at risk; (**C**) KM curve of the RI rate grouped by the virulence of infected pathogens with the numbers of patients at risk.

During the follow-up period, RI were documented in 10 patients (four in the EAR group, two in the ISR group and four in the conservative treatment group). Among these RI patients eight of them were re-admitted to our department for a secondary operation or conservative treatment, while the other two patients were treated in a local hospital with insufficient information about their treatment. Statistical analysis revealed that the RI rate in the ISR group was significantly lower than the EAR group and conservative treatment group (*p* < 0.001 and *p* < 0.005, respectively; see Figure 4A). However, the RI rate between the EAR and the conservative treatment group was not significantly different (*p* = 0.054). The estimated overall RI-free rate of AGI patients was 75.5%, 68.6%, and 58.8% at 1, 3 and 5 years after treatment.

We also investigated whether different conduits used in the ISR or EAR procedures were related to the RI rate. Autologous vein was used for bypass conduits in 20 of 39 AGI patients, while rifampicin-soaked ePTFE vascular grafts were used in eight patients and normal ePTFE vascular grafts were used in the remaining patients. The sub-group analysis revealed that RI rates in the autologous vein group was not significantly different from the rifampicin-soaked and normal ePTFE vascular grafts groups (*p* = 0.874 and *p* = 0.249, respectively, see Figure 4B).

Furthermore, statistical analysis also revealed that AGI patients infected with high virulence pathogens were more likely to have RI than patients with low virulence pathogens, despite no significant difference (*p* = 0.211, see Figure 4C). Since the number of enrolled and sub-group patients were relatively small, the reliability of Kaplan-Meier survival curves may be low. A survival table was also used to display the RI and graft thrombosis situation of AGI patients, which was consistent with the statistical analysis result of Kaplan-Meier survival curves (see Table 6).

#### 3.2.3. Primary and Secondary Graft Patency

Six cases of late thrombosis were documented during the follow-up period, including cases in the ISR and EAR groups. Of the six cases, four received secondary intervention because of the presence of lower limb ischemia and two patients received major amputation despite performing reintervention surgery. The limb salvage rates were 96% at 1 year and 93% at 3 and 5 years.

**Figure 5:**
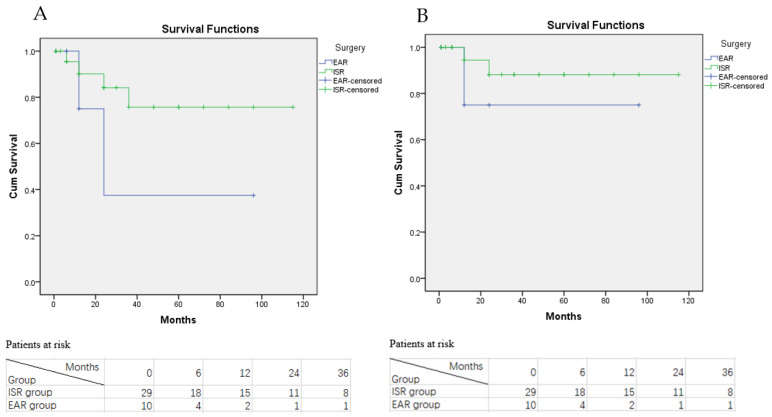
Kaplan-Meier survival curve of graft patency grouped by different treatment option. (**A**) KM curve of primary graft patency of ISR and EAR group with numbers of patients at risk; (**B**) KM curve of secondary graft patency of ISR and EAR group with numbers of patients at risk.

The estimated primary graft patency of ISR group at 1 year was 75%, while the corresponding primary graft patency of the EAR group was 90.2% at 1 year. The log-rank test revealed no difference in primary graft patency between the ISR and EAR groups (*p* = 0.163, see Figure 5A). Similarly, the estimated secondary graft patency of the ISR (75% at 1 year) and EAR (94.4% at 1 year) groups was also not significantly different (*p* = 0.344, see Figure 5B).

**Figure 6:**
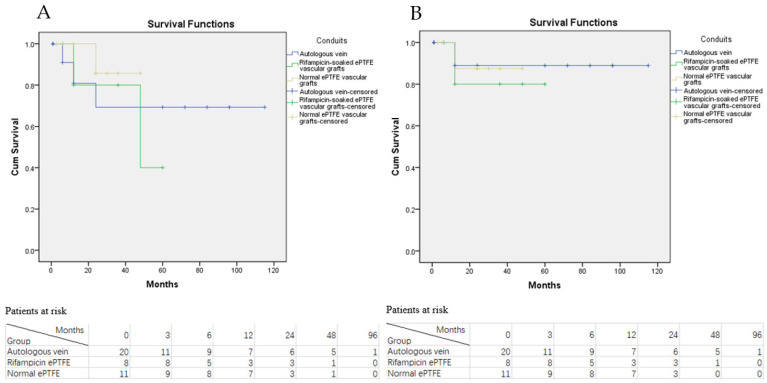
Kaplan-Meier survival curve of graft patency grouped by different bypass graft conduits. (**A**) KM curve of primary graft patency of different bypass graft conduits with the numbers of patients at risk; (**B**) KM curve of secondary graft patency of different bypass graft conduits with the numbers of patients at risk.

Furthermore, the estimated primary and secondary graft patency of AGI patients grouped by different conduits was also investigated. Sub-group analysis results suggest that the estimated primary graft patency of the autologous vein was not significantly higher than that of the rifampicin-soaked ePTFE and the normal ePTFE vascular grafts (*p* = 0.652 and *p* = 0.165, respectively, see Figure 6A). Additionally, the secondary graft patency of autologous vein was also not significantly different from the rifampicin-soaked ePTFE and the normal ePTFE vascular grafts based on the results of the log-rank test (*p* = 0.606 and *p* = 0.915, see Figure 6B).

#### 3.2.4. Overall Mortality

**Figure 7:**
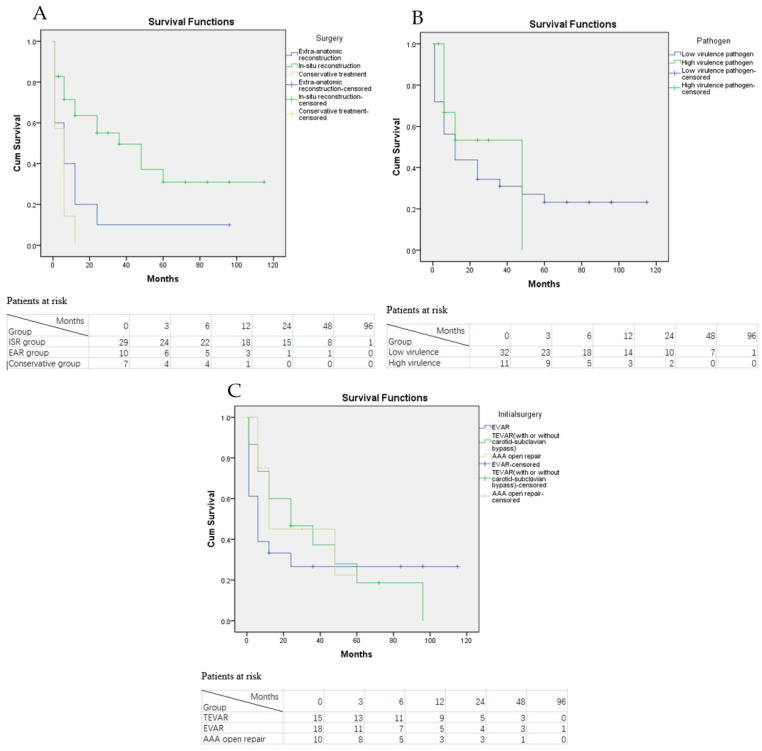
Kaplan-Meier survival curve of overall mortality. (**A**) KM curve of overall mortality grouped by treatment option with the numbers of patients at risk; (**B**) KM curve of overall mortality grouped by the virulence of pathogens with the numbers of patients at risk; (**C**) KM curve of overall mortality grouped by the initial procedure prior to AGI with the numbers of patients at risk.

In total, nine late deaths were documented on follow-up. The reason was respiratory failure in two patients, chronic kidney dysfunction in one patient, heart failure in one patient, myocardial infarction in one patient, aortic dissection in one patient, gastrointestinal bleeding in one patient, cerebral infarction in one patient, and unknown in one patient. The estimated overall survival rate for all patients was 58% at 1 year, 32% at 3 years, and 18% at 5 years. The Kaplan–Meier survival rates of the ISR group were 63%, 49%, and 31% at 1, 3, and 5 years, respectively (see Figure 7A), and those of the EAR groups were 20% at 1 year and 10% at 3 and 5 years. The log-rank test showed that the overall mortality rate in the ISR group was significantly lower than those in the EAR and conservative treatment groups (*p* = 0.01 and *p* < 0.001). The overall mortality did not differ significantly between the EAR and conservative treatment groups (*p* = 0.124).

The effect of infected pathogens and initial procedures on overall mortality was also assessed. The Log-rank test revealed that despite the fact that the overall mortality of AGI patients with high virulence pathogens was higher than that of patients with low virulence pathogens, no significant difference in the overall mortality was observed between the two groups (*p* = 0.350, see Figure 7B). Sub-group analysis also revealed that AGI patients who underwent AAA open repair as their initial procedures had lower overall mortality than patients who underwent TEVAR or EVAR; however, no significant difference was observed among the three groups (*p* = 0.573, 0.097 and 0.782, respectively, see Figure 7C).

#### 3.2.5. Duration of Antibiotic Therapy and Types of Antibiotics Used

The mean durations of antibiotic therapies in the EAR, ISR, and conservative treatment groups were 17.5 ± 13.6, 14.3 ± 10.9, and 5.0 ± 1.4 months, respectively, with the antibiotic therapy administered for significantly longer in the ISR and EAR groups than in the conservative treatment group (*p* < 0.05). The total types of antibiotics used during treatment were 2.8 ± 1.4, 3.0 ± 1.1, and 2.7 ± 0.4 in EAR, ISR, and conservative treatment groups, respectively, and no significant between-group differences were identified. The average duration of antibiotic therapy was 12.7 ± 10.4, and 13.1 ± 11.4 months in patients with and without post-operative RI, respectively (*p* = not significant), however, a significantly greater number of antibiotics were used in patients without RI (2.3 ± 0.9 vs. 3.4 ± 0.8, *p* < 0.001).

## 4. Discussion

AGI is a challenging complication with high perioperative and overall mortality [2,3,6,9,10]. To treat the vascular prosthetic infection, a complete removal of the infected graft combined with EAR (mostly axillofemoral bypass) was considered the standard treatment. However, because of the high perioperative complication rates and overall mortality associated with EAR, ISR was introduced by the University of Texas-Houston group to treat AGI in the late 1980s, and it has since become the treatment of choice owing to its safety and durability [11]. In our study, ISR had an acceptable early mortality, complication-related morbidity, primary graft patency, a significantly lower RI rate, and lower overall mortality rate than EAR and conservative treatments. Our study confirms that ISR is a safe treatment choice in cases of AGI, which is in line with the conclusion drawn by earlier studies [5,6,7,9,12,13,14].

PCT is a precursor of calcitonin, which is produced by the thyroid gland. The PCT level is known to increase under several circumstances, such as bacterial infection, major trauma or surgery, and malignant tumors. A systematic review conducted in 2017 concluded that the PCT level is not associated with mortality, RI, mechanical ventilation, or duration of antibiotic therapy and that it has no prognostic value in cases of septic or severe septic shock [15]. However, PCT evaluation yields high sensitivity and specificity for infection and is hence valuable for early diagnosis and informing treatment decisions [16,17]. In addition, a study showed that a CRP level >50 mg/L is associated with mortality in patients with AGI, and CRP has also been reported as a reliable factor associated with an increased RI rate in patients with stent infection [18,19]. Thus, we believe that increased levels of inflammatory chemical indicators in AGI patients, such as CRP, IL-6, and PCT, may indicate a poor prognosis and prompt surgeons toward proactive management. No previous study reported the potential association between peak PCT level and 30-day mortality. Although univariate regression results revealed the potential association between the peak PCT level and 30-day mortality, univariate and multivariate regression analysis results investigating this relationship have been inconsistent. We assume that the contradictory results may be attributed to the existence of confounding factors and the limited number of enrolled patients, therefore, more large-scale and prospective studies may be necessary.

Our study also demonstrated a higher 30-day mortality rate in patients requiring emergency intervention than in patients who underwent elective surgery, which is in line with previous studies [6,7,9,19]. A few studies have also reported age, chronic kidney dysfunction, higher CRP levels, and coronary artery disease as risk factors associated with a poorer prognosis and higher mortality [19,20,21]. However, the aforementioned risk factors were not associated with mortality in our cohort.

While current practice includes the immediate administration of antibiotics to patients with AGI diagnosed with an established vascular prosthetic infection, a consensus on the best duration for oral antibiotic administration after surgery or conservative treatment and the time for hospital discharge remains lacking. In our study, although RI and the duration of antibiotic therapy were not associated, patients receiving ISR and EAR received antibiotic therapy longer than those who received conservative treatment. This could be attributed to the longer survival of patients with ISR and EAR. Furthermore, patients without RI received more types of antibiotics during treatment, which indicates the need for regular blood or abscess cultures to establish evidence of bacteria and adjust antibiotic therapy depending on the results. We recommend that the duration of antibiotic therapy be decided considering the patient’s clinical symptoms and auxiliary examination results, that blood culture tests be conducted periodically during the treatment and follow-up, and that the duration of antibiotic therapy be at least 3–6 months. For patients with AGI experiencing RI, lifetime antibiotic therapy might be required.

Various vascular conduits are used in ISR and EAR surgery, including autologous vein, cryopreserved allografts, silver-coated grafts, rifampicin bonded polyester grafts, and bovine pericardium. In our cohort, autologous vein, rifampicin-soaked, and normal ePTFE vascular grafts were used as conduits in ISR or EAR surgery. Statistical analysis demonstrated no significant differences in the patency of primary and secondary graft conduits. Furthermore, there was no significant difference in the RI rates between these conduits. A few studies have demonstrated the reputation of autologous veins for resisting infection and possessing acceptable graft patency rates in AGI patients [22,23,24]. However, nearly all of these studies, like the present one, were retrospective and observational, which posed problems concerning interstudy heterogeneity and selection bias. In addition, a recent meta-analysis conducted by Batt et al. found that, despite autologous vein having the lowest RI rate of 6%, no significant differences were observed between autologous vein and other common bypass conduits [25]. It is also revealed that the RI rate of autologous veins shows a negative correlation with age and prosthetic-duodenal fistula. In our cohort, the median age of patients was 70, and there were many patients suffering from an AEnF; thus, autologous veins may not be the best bypass conduit choice for these patients. The RI rate of autologous vein was similar to PTFE vascular grafts. As for the graft patency rate, our research demonstrated that there was no significant difference in the primary and secondary graft patency of autologous vein when compared to PTFE vascular grafts. However, a previous study showed that only 2% of autologous veins had graft occlusion issues and had significant advantages over other common bypass conduits, except for silver-coated polyester grafts [22]. The discrepancy in our results may be attributed to the fact that the total and sub-group numbers of enrolled patients in our cohort were limited. Furthermore, our research was a retrospective and non-randomized study with only one participating institution, which may cause selection bias and affect the statistical analysis.

Our study showed that patients who underwent conservative treatment or total graft removal plus EAR had a higher risk of RI and overall mortality than patients who underwent total graft removal plus ISR. Furthermore, overall mortality and RI rates did not differ between patients receiving conservative treatment or EAR only. This could be attributed to the poor general status and low life expectancy of these patients, and our data suggest that RI is more likely to occur in these patients. Our study failed to establish whether the initial procedures of AGI patients have an influence on a patient’s prognosis. However, performing ISR or EAR surgery for patients who underwent TEVAR previously may be more challenging and take more time. This may affect the prognosis of AGI patients, and thus, more high-quality studies are necessary. A few studies indicated that partial graft removal is acceptable when the infected region is small and localized, with the remaining graft being stable [26,27,28]. However, partial graft removal should only be considered when patients are not eligible for total graft removal plus ISR. An earlier study also reported that partial graft removal is associated with a risk of RI [6]. Although ISR is now the first-line treatment for patients with AGI, EAR remains an important treatment choice for AGI. Oderich et al. and Heinola et al. indicated that EAR is suitable for patients with large peri-graft abscesses or MRSA infections [7]. Although EAR is associated with higher mortality and RI rates, it remains the preferred choice for AGI patients.

The clinical symptoms, prognosis, and hence the treatment strategy also vary depending on the infecting pathogenic bacteria. *Bandyk* et al. used ISR with rifampin-soaked gelatin-sealed polyester grafts in patients with vascular prosthetic infection caused by low-virulence pathogens, such as *Staphylococcus epidermidis* or *Salmonella*, and reported good outcomes with low mortality and RI rates [29]. However, in the case of infections with high-virulence pathogens, such as MRSA or other multiple resistant bacteria, the prognosis is poor, and ISR may be unsafe [30]. Our data suggests that patients with high-virulence pathogen infections had severe clinical presentations, higher RI rates, and a poorer prognosis than patients with low-virulence pathogen infections, although no statistically significant difference was observed between these groups. Despite no positive findings, it is important to note that the negative results may be due to the small number of enrolled patients in this study as well as the virulence of pathogens in AGI patients, which influence the treatment choice. For patients infected with high virulence pathogens, EAR may be considered first-line treatment, and the duration of antibiotics would be extended because ISR may no longer be the best option for AGI patient’s and our data suggests that high-virulence pathogens are more likely to cause RI.

At our institution, the patients’ general status, clinical presentations, and life expectancy are considered in making the decision to administer a surgical intervention. ISR is our first choice for surgical treatment of AGI. EAR is considered only if the patient has a large abscess that precludes satisfactory results with ISR or infection with unmanageable multiple resistant bacteria. In addition, we have set several criteria that patients with AGI must meet before hospital discharge, including negative CT findings, normal body temperature for at least 14 days, and 3 consecutive negative blood culture results. Lifelong follow-up is preferred if possible, given that the risk of RI is reported to increase with a prolonged follow-up period [6].

Our study had several limitations. First, it was a retrospective and non-randomized study in which only one institution participated, which may cause selection bias and affect the statistical analysis. Furthermore, the number of overall and sub-group enrolled patients was relatively small. However, its strengths lie in the relatively long follow-up period and several positive findings regarding the factors that affect the prognosis of AGI patients.

## 5. Conclusions

We found that ISR had a lower RI rate and better overall survival rates than EAR and conservative treatment, and ISR may be a better choice for patients with AGI than EAR and conservative treatment. We also evaluated several factors that may affect the prognosis of AGI patients, and it was revealed that the RI rate and primary and secondary graft patency of autologous veins were not better than ePTFE vascular grafts. Furthermore, statistical analysis revealed that high-virulence pathogens may be related to higher RI and overall mortality rates, despite the fact that no significant difference was found. Initial procedures prior to AGI were also found to have no influence on the prognosis of AGI patients. Due to the fact that the overall incidence of AGI was relatively low and the number of enrolled patients in our study was small, more large-scale, high-quality, multicenter randomized controlled trials are necessary.

## Figures and Tables

**Table 1:** Baseline information and clinical presentation of patients with AGI.

Baseline Information and Clinical Presentations	n (%)
**Demographics**	
Male, n (%)	30 (69%)
Median age(IQR)	70 (57–78)
**Pre-operative comorbidities, n(%)**	
Hypertension	31 (72%)
Smoking/Past smoking history	25 (58%)
Diabetes mellitus	17 (39%)
Chronic obstructive pulmonary disease	11 (25%)
Hypoproteinemia	10 (23%)
Coronary artery disease	6 (13%)
Chronic kidney failure	5 (11%)
**Clinical presentations**	
Sepsis(fever, leukocytosis and bacteremia)	29 (67%)
Abdominal/back pain	23 (53%)
Peri-graft/retroperitoneal abscess	21 (48%)
Graft duodenal fistula	10 (23%)
Acute major bleeding	8 (18%)
Lower-limb ischemia	5 (11%)
Pseudoaneurysms	3 (6%)
**Highest CRP level during hospital stay**	
>100 mg/L	29 (68%)
<100 mg/L	11 (25%)
N/A	3 (6%)
**Highest PCT level during hospital stay**	
>1 ng/mL	31 (72%)
<1 ng/mL	9 (22%)
N/A	3 (6%)
**Surgery type**	
Selective surgery	32 (75%)
Emergency surgery	11 (25%)
**Total**	**43**

Data are presented as n (%) or median (interquartile range, IQR) unless stated otherwise. 35 patients presented with >1 pre-operative comorbidity, and 28 patients presented with >1 clinical presentation. N/A: Not applicable.

**Table 2:** Initial treatment of previous vascular diseases of AGI patients.

Initial Treatment	n	%
EVAR	18	42
TEVAR	13	30
AAA open repair	10	23
TEVAR + carotid-subclavian bypass	2	5
**Total**	**43**	**100**

EVAR: endovascular aortic aneurysm repair; TEVAR: thoracic endovascular aortic aneurysm repair; AAA: abdominal aortic aneurysm.

**Table 3:** Patients’ microbiological culture results.

Pathogen	n	%
**Gram-Positive bacterium**		
*Staphylococcus aureus*	15	34
MRSA	4	9
*Streptococcus*	3	6
*Enterococcus*	3	6
**Gram-negative bacterium**		
*Escherichia coli*	12	27
*Klebsiella pneumoniae*	9	20
*Pseudomonas aeruginosa*	5	11
*Enterobacter cloacae*	3	6
*Salmonella*	1	2
Fungus infection	2	6
**Polymicrobial infection**	17	39
**Negative culture results**	6	13
**Total**	**43**	**100**

37 patients had positive bacterial culture results and 6 patients had negative bacterial culture results, >1 pathogen was cultured in 17 patients. MRSA: Methicillin-resistant staphylococcus aureus.

**Table 4:** Univariate analysis of risk factors associated with 30-day mortality.

Variables	Survived	Deceased	*p*
**Demographics**			
Male	23	7	0.71
Age (Mean ± SD)	70.78 ± 7.05	69.55 ± 10.39	0.66
**Pre-operative comorbidity**			
Hypertension	22	9	0.46
Smoking	19	6	1.00
Diabetes mellitus	12	5	0.73
COPD	8	3	1.00
Hypoproteinemia	6	4	0.25
Coronary artery disease	4	2	0.64
CKD	3	2	0.59
**Clinical Presentations**			
Sepsis	21	8	1.00
Abdominal/back pain	17	6	1.00
Abscesses	14	7	0.31
Graft duodenal fistula	5	5	0.09
Acute major bleeding	5	3	0.40
Lower limb ischemia	2	3	0.10
Pseudoaneurysms	1	2	0.15
**Highest CRP level**			
>100 mg/L	19	10	0.23
**Highest PCT level**			
>1 ng/mL	20	11	0.04
**Surgery type**			
Emergency surgery	5	6	0.02
**Total**			**43**

3 patients had no CRP and PCT examination results, hence were not included in the univariate analysis for the relationship between CRP/PCT level and 30-day mortality. SD: standard error; COPD: chronic obstructive pulmonary disease; CKD: chronic kidney disease; CRP: C-reactive protein; PCT: procalcitonin.

**Table 5:** Multivariate analysis of potential predictors related to 30-day mortality.

Variables	*p*	95% CI
Emergency surgery	0.03	1.25–26.57
Highest PCT level	0.38	0.28–29.56
Highest CRP level	0.08	0.78–60.12
Graft duodenal fistula	0.21	0.56–13.95
**Total**	**43**	

Only Emergency surgery was associated with 30-day mortality in the multivariate analysis. PCT: procalcitonin; CRP: C-reactive protein.

**Table 6:** Survival table of the RI and graft thrombosis situation in AGI patients.

Treatment Options	Time (Months)	Proportion Surviving for RI	Cumulative Proportion Surviving at the End of RI Interval	Proportion Surviving for Graft Thrombosis	Cumulative Proportion Surviving at End of Graft Thrombosis Interval
EAR	0	0.75	0.75	1.00	1.00
	10	0.71	0.54	0.71	0.71
	20	1.00	0.54	0.50	0.36
	30	1.00	0.54	1.00	0.36
	40	1.00	0.54	1.00	0.36
	50	1.00	0.54	1.00	0.36
	60	1.00	0.54	1.00	0.36
	70	1.00	0.54	1.00	0.36
	80	1.00	0.54	1.00	0.36
	90	0.00	0.00	1.00	0.36
ISR	0	0.96	0.96	0.96	0.96
	10	1.00	0.96	0.94	0.94
	20	1.00	0.96	0.93	0.84
	30	1.00	0.96	0.90	0.75
	40	1.00	0.96	1.00	0.75
	50	1.00	0.96	1.00	0.75
	60	0.82	0.78	1.00	0.75
	70	1.00	0.78	1.00	0.75
	80	1.00	0.78	1.00	0.75
	90	1.00	0.78	1.00	0.75
	100	1.00	0.78	1.00	0.75
	110	1.00	0.78	1.00	0.75
Conservative	0	0.45	0.45	NA	NA
treatment	10	0.00	0.00	NA	NA

Patients in conservative treatment groups were not included in the graft thrombosis analysis. Statistical analysis revealed that the RI rate among ISR, EAR, and conservative treatment group was significantly different (*p* < 0.001), while no difference was observed in the graft patency between the ISR and EAR group. RI: re-infection; ISR: in-situ reconstruction; EAR: extra-anatomic reconstruction (*p* = 0.32); NA: not applicable.

## Data Availability

The data presented in this study are available on request from the corresponding author. The data are not publicly available due to ethics and privacy reasons.
